# Hilar lymphadenopathy, a novel finding in the setting of coronavirus disease (COVID-19): a case report

**DOI:** 10.1186/s13256-020-02452-3

**Published:** 2020-08-09

**Authors:** Mohsin Sheraz Mughal, Rameez Rehman, Ramy Osman, Nathan Kan, Hasan Mirza, Margaret H. Eng

**Affiliations:** 1 Monmouth Medical Center, An Affiliate of RWJ/Barnabas Health System, 300 2nd Avenue, Long Branch, NJ 07740 USA; 2 Beth Israel Deaconess Medical Center/Harvard Medical School, Boston, MA USA

**Keywords:** Coronavirus, Lymphadenopathy, Viral pneumonia, COVID-19

## Abstract

**Background:**

As the outbreak of coronavirus disease 2019 (COVID-19) has progressed, computed tomography has emerged as an integral part of the diagnosis alongside reverse transcriptase–polymerase chain reaction assays. Frequently encountered imaging findings include peripheral airspace consolidations; bilateral ground-glass opacities; and, less commonly, cavitation. Hilar lymphadenopathy is a rarely reported finding in the setting of COVID-19.

**Case presentation:**

A 73-year-old Caucasian woman presented to our hospital with fever and fatigue. She had a maximum body temperature of 102.3 °F with lymphopenia and thrombocytopenia. She was diagnosed with severe acute respiratory syndrome coronavirus 2 infection on the basis of a positive result from a reverse transcriptase–polymerase chain reaction of a nasopharyngeal swab sample. Contrast-enhanced chest computed tomography revealed multifocal, subpleural ground-glass opacities with nodular consolidations bilaterally. Computed tomography also demonstrated atypical bilateral hilar lymphadenopathy, a rarely reported imaging feature of COVID-19. Chest computed tomography 1 month before the presentation did not show focal consolidations or lymphadenopathy. This indicated that the findings were due to the patient’s severe acute respiratory syndrome coronavirus 2 infection. She received 5 days of oral hydroxychloroquine and experienced resolution of her symptoms.

**Conclusion:**

Chest computed tomography has been used extensively to diagnose and characterize the distinguishing radiological findings associated with viral pneumonia. It has emerged as an integral part of the diagnosis of COVID-19 alongside reverse transcriptase–polymerase chain reaction assays. Clinicians must be aware of uncommon clinical and radiological findings in order to diagnose this entity. Hilar lymphadenopathy is commonly seen with fungal infections, mycobacterial infections, and sarcoidosis. An extensive literature review found that bilateral hilar lymphadenopathy has not been reported in the setting of COVID-19. More data are needed to establish the clinical impact of this novel finding.

## Introduction

The World Health Organization declared coronavirus disease 2019 (COVID-19) a pandemic in March 2020. Though the incidence of the disease has drastically dropped in China, it is rising worldwide [1]. Coronavirus is an encapsulated ribonucleic acid (RNA) virus, a novel coronavirus named severe acute respiratory syndrome coronavirus 2 (SARS-CoV-2), which was identified as the culprit of a cluster of lower respiratory tract illnesses in Wuhan, China. A study suggested that it has two strains: type S and type L [2]. The understanding of the disease, its transmission, and its treatment are still evolving. COVID-19 can range from being asymptomatic to a wide variety of severe symptoms [3]. Several observational studies suggested that fever, malaise, dry cough, and dyspnea are the most common presenting symptoms [4]. As the outbreak of COVID-19 has progressed, so have the methodologies used in its diagnostic workup. A chest computed tomographic (CT) scan has quickly emerged as an integral part of the diagnosis alongside reverse transcriptase–polymerase chain reaction (RT-PCR) assays [5]. The most commonly reported CT findings in patients with COVID-19 are bilateral ground-glass opacities [6]. In another study, CT manifestations were peripheral airspace consolidations in one-third of the cases. Less common findings include pleural effusion, pericardial effusion, cavitation, air bronchograms, and pneumothorax [7]. These data are helpful for clinicians to gain an understanding of a wide spectrum of imaging findings in patients with COVID-19. Hilar lymphadenopathy is a common radiological finding associated with fungal infections, mycobacterial infections, and sarcoidosis. However, it is rarely seen in viral pneumonia. Because the COVID-19 pandemic is affecting healthcare and economic systems worldwide, it is imperative to detect the disease earlier in the course before complications involving acute hypoxic respiratory failure warranting invasive mechanical ventilation arise. Characteristic CT scan findings, alongside RT-PCR and antibody testing, help clinicians diagnose COVID-19. None of these modalities can be used as a single tool to diagnose SARS-CoV-2 infection. An extensive literature review found that acute bilateral hilar lymphadenopathy has not been reported in the setting of COVID-19 [6, 8]. It is important to report atypical imaging findings to establish their frequency and association with disease severity and outcomes.

## Case presentation

**Fig. 1:**
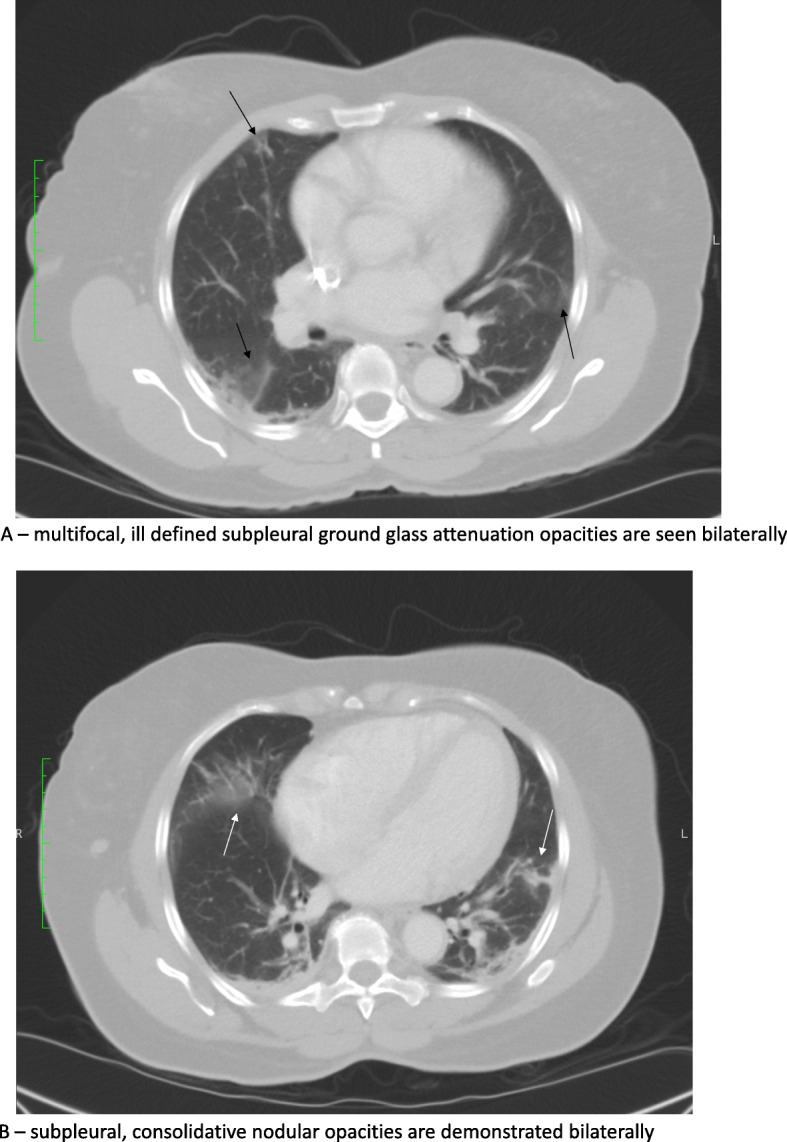
Contrast-enhanced chest computed tomography reveals multifocal, subpleural ground-glass attenuation opacities (arrow) (**a**) with nodular consolidations bilaterally (arrow) (**b**)

**Fig. 2:**
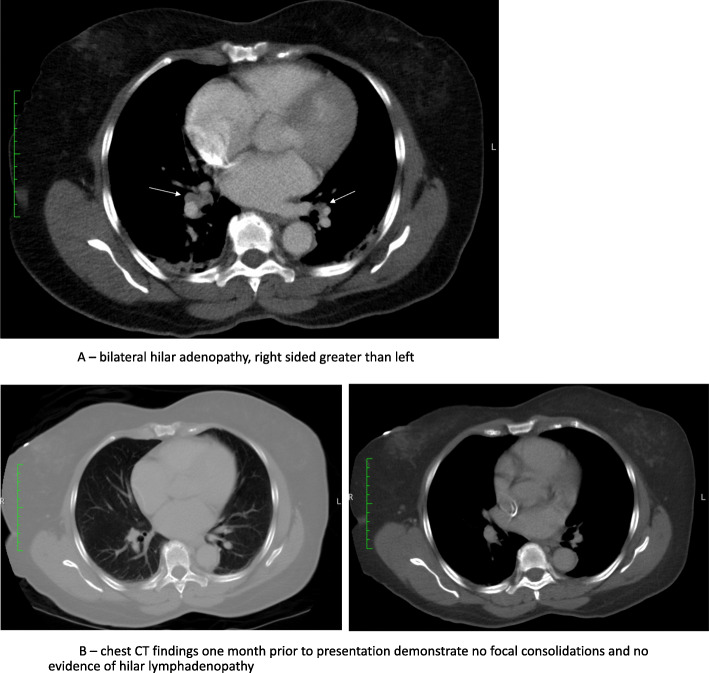
**a** Atypical bilateral hilar lymphadenopathy (arrow), a novel finding of coronavirus disease 2019. **b** Chest computed tomography 1 month before presentation demonstrates no focal consolidations and no lymphadenopathy

A 73-year-old Caucasian woman with a past medical history of hypertension, hyperlipidemia, pulmonary embolism, and rheumatoid arthritis came to our emergency department with complaints of fever, chills, generalized weakness, and decreased appetite of 1 day’s duration. Her home medication list included amlodipine 10 mg, pantoprazole 40 mg, rivaroxaban 20 mg, and omega-3 polyunsaturated fatty acid 1000 mg. The patient had a remote history of rheumatoid arthritis, for which she was not taking any medication. She had quit smoking almost 30 years ago and admitted to one or two glasses of alcohol consumption occasionally. In the emergency department, she was febrile with a maximum body temperature of 102.3 °F. Her blood pressure was 157/73 mmHg, heart rate was 81 beats/minute, respiratory rate was 16 breaths/minute, and pulse oxygen saturation was 96% on room air. At admission, she was alert and oriented and did not seem to be in any distress. Her pulse was regular; her heart sounds (S1, S2) were audible without any murmur or additional heart sounds; and no lower extremity edema was observed during her physical examination. Her lungs were grossly clear with equal air entry without any wheeze or rhonchi. Her abdomen was soft, and bowel sounds were present. Her hematological workup showed leukopenia (white blood cell count, 3400/mm^3^), anemia (hemoglobin, 10.5 g/dl), thrombocytopenia (platelets, 163,000/mm^3^), and elevated inflammatory markers including C-reactive protein (66.8 mg/L) and erythrocyte sedimentation rate (74 mm/hour). The result of multiplex respiratory viral polymerase chain reaction (PCR) was negative for respiratory tract viral infections. Liver function tests, including aspartate aminotransferase (26 U/L) and alanine aminotransferase (ALT 8 U/L), and a basic metabolic panel, including blood urea nitrogen (16 mg/dl) and creatinine (1.03 mg/dl), were within the normal ranges. The result of a nasopharyngeal (NP) swab sample for SARS-CoV-2 was positive by RT-PCR, and a diagnosis of COVID-19 was established. Blood cultures and sputum culture did not show any bacterial or fungal growth. The patient started developing a dry cough and respiratory distress requiring supplemental oxygen via a nasal cannula (up to 6 L/minute) on the second day of admission with sporadic rhonchi detected by physical examination. Contrast-enhanced chest CT revealed multifocal, subpleural ground-glass opacities with nodular consolidations bilaterally (Fig. 1a, b). A CT scan also demonstrated atypical bilateral hilar lymphadenopathy, a rarely reported CT finding in COVID-19 (Fig. 2a). Chest CT one month before the current presentation demonstrated no focal consolidations or lymphadenopathy (Fig. 2b). The patient received hydroxychloroquine (HCQ) 200 mg and azithromycin 500 mg orally for 5 days alongside supportive treatment with acetaminophen and supplemental oxygenation via nasal cannula. Her fever resolved, and her respiratory status improved. At discharge, she was breathing without any distress on room air.

## Discussion

Chest CT has been used extensively not only to diagnose but also to characterize the distinguishing radiological findings associated with viral pneumonia. SARS-CoV-2 is diagnosed by COVID-19 symptomatology and RT-PCR with NP swabs. As the outbreak of COVID-19 has progressed, CT alongside RT-PCR has been used broadly to diagnose COVID-19 [9]. There is convincing evidence that viral load is high in NP samples, and RT-PCR can have false-positive and false-negative results. If there is a high clinical suspicion, then repeat RT-PCR with resampling from another site is recommended [10]. There have been patients with a negative result of RT-PCR but whose CT scan was suggestive of viral pneumonia. Later, those patients had positive test results for SARS-CoV-2 [11, 12]. Yan Li and Liming Xia suggested that CT scans can be used as a rapid diagnostic tool to diagnose COVID-19 on the basis of a low rate of missed diagnoses [13]. This argues in favor of chest imaging earlier in the course with clinical suspicion of viral pneumonia. Frequently encountered imaging findings include peripheral airspace consolidations and bilateral ground-glass opacities; less common associations include cavitation and air bronchograms. To our knowledge, hilar lymphadenopathy has not been reported in the setting of COVID-19. RT-PCR of the NP swab sample is the preferred and recommended screening test worldwide. However, false-negative results may occur because of inadequate viral load or impaired sampling techniques. In these circumstances, chest CT scans and SARS-CoV-2 immunoglobulin M antibody testing can significantly aid in the diagnostic workup. CT scan findings can vary on the day of imaging, and some studies even suggested obtaining a repeat CT scan to rule out worsening of the disease [14]. Clinicians must be aware of rare clinical and radiological findings in order to diagnose this entity. In the absence of effective antiviral therapy and persistent evidence-based guidelines, HCQ and zinc were initially used for COVID-19. Interleukin-6 inhibitors are used against the hyperinflammatory state because of the proposed cytokine storm syndrome. Immunoglobulin G antibodies against SARS-CoV-2 start developing after 2 weeks of disease onset, and convalescent plasma from the recovered patient population is being studied in compassionate trials at different centers. The efficacy of these medications and therapeutic interventions is yet to be established. Calcified lymph nodes have been reported in the late stages of alphaherpesvirus pneumonia [15]. However, bilateral hilar lymphadenopathy has not been reported in the setting of COVID-19. Reporting and recognizing the rare imaging findings will help clinicians to understand their frequency and association with the disease. Follow-up imaging should be pursued to evaluate the persistence or resolution of hilar lymphadenopathy. More information with long-term follow-up is required to establish the importance and clinical implications of our findings.

## Conclusion

Chest CT has been used extensively to diagnose and characterize the distinguishing radiological findings associated with viral pneumonia. It has emerged as an integral part of the diagnosis of COVID-19 alongside RT-PCR. Clinicians must be aware of uncommon clinical and radiological findings in order to diagnose this entity. Hilar lymphadenopathy is commonly seen with fungal infections, mycobacterial infections, and sarcoidosis. In an extensive literature review, bilateral hilar lymphadenopathy was not reported in the setting of COVID-19. Recognizing rare imaging findings will help clinicians understand their frequency and association with disease severity. Follow-up imaging should be pursued to evaluate the persistence or resolution of hilar lymphadenopathy in patients with COVID-19.

## Data Availability

Not applicable.
